# Association of Upper Gastrointestinal Surgery of Great Britain and Ireland (AUGIS)/Perioperative Quality Initiative (POQI) consensus statement on intraoperative and postoperative interventions to reduce pulmonary complications after oesophagectomy

**DOI:** 10.1093/bjs/znac193

**Published:** 2022-08-24

**Authors:** Pritam Singh, James Gossage, Sheraz Markar, Philip H Pucher, Alex Wickham, Jonathan Weblin, Swathikan Chidambaram, Alexander Bull, Oliver Pickering, Monty Mythen, Nick Maynard, Mike Grocott, Tim Underwood, AUGIS/POQI Pulmonary Consensus Group, M Mythen, N Maynard, M Grocott, T Underwood, O Pickering, P Singh, S Markar, D Levett, C Alan, N Tewari, F Noble, K Moorthy, M Oliver, S Chidambaram, A Wickham, J Gossage, P Pucher, A Bull, J Sultan, F Huddy, S Davies, J Weblin, M West

**Affiliations:** Department of General Surgery, Royal Surrey NHS Foundation Trust, Surrey, UK; Department of Upper Gastrointestinal Surgery, Guy’s and St Thomas’ Hospitals NHS Trust, London, UK; Department of Upper Gastrointestinal Surgery, Oxford University Hospitals NHS Foundation Trust, Oxford, UK; Department of Molecular Medicine and Surgery, Karolinska Institute, Solna, Sweden; Department of Upper Gastrointestinal Surgery, Portsmouth Hospitals University NHS Trust, Portsmouth, UK; Department of Anaesthesia, Imperial College Healthcare NHS Trust, London, UK; Department of Physiotherapy, Queen Elizabeth Hospital Birmingham, Birmingham, UK; Department of General Surgery, Imperial College Healthcare NHS Trust, London, UK; Department of Upper Gastrointestinal Surgery, Guy’s and St Thomas’ Hospitals NHS Trust, London, UK; School of Cancer Sciences, University of Southampton Faculty of Medicine, Southampton, UK; Centre for Anaesthesia, Critical Care and Pain Management, University College London Hospitals NHS Foundation Trust, London, UK; Department of Upper Gastrointestinal Surgery, Oxford University Hospitals NHS Foundation Trust, Oxford, UK; NIHR Southampton Respiratory Biomedical Research Unit, University Hospital Southampton NHS Foundation Trust, Southampton, UK; School of Cancer Sciences, University of Southampton Faculty of Medicine, Southampton, UK

## Abstract

**Background:**

Pulmonary complications are the most common morbidity after oesophagectomy, contributing to mortality and prolonged postoperative recovery, and have a negative impact on health-related quality of life. A variety of single or bundled interventions in the perioperative setting have been developed to reduce the incidence of pulmonary complications. Significant variation in practice exists across the UK. The aim of this modified Delphi consensus was to deliver clear evidence-based consensus recommendations regarding intraoperative and postoperative care that may reduce pulmonary complications after oesophagectomy.

**Methods:**

With input from a multidisciplinary group of 23 experts in the perioperative management of patients undergoing surgery for oesophageal cancer, a modified Delphi method was employed. Following an initial systematic review of relevant literature, a range of anaesthetic, surgical, and postoperative care interventions were identified. These were then discussed during a two-part virtual conference. Recommendation statements were drafted, refined, and agreed by all attendees. The level of evidence supporting each statement was considered.

**Results:**

Consensus was reached on 12 statements on topics including operative approach, pyloric drainage strategies, intraoperative fluid and ventilation strategies, perioperative analgesia, postoperative feeding plans, and physiotherapy interventions. Seven additional questions concerning the perioperative management of patients undergoing oesophagectomy were highlighted to guide future research.

**Conclusion:**

Clear consensus recommendations regarding intraoperative and postoperative interventions that may reduce pulmonary complications after oesophagectomy are presented.

## Teaser

Pulmonary complications are the most common morbidity after oesophagectomy, contributing to mortality and prolonged postoperative recovery, and have a negative impact on short- and long-term health-related quality of life. Although numerous perioperative interventions to reduce the risk of pulmonary complications have been presented, significant variation in practice exists. Through a modified Delphi process, this Association of Upper Gastrointestinal Surgery of Great Britain and Ireland/Perioperative Quality Initiative, involving a multidisciplinary group of over 20 experts, has delivered clear evidence-based recommendations regarding intraoperative and postoperative care that can reduce pulmonary complications.

## Introduction

Pulmonary complications are the most common morbidity after oesophagectomy, contributing to mortality and prolonged postoperative recovery, and have a negative impact on short- and long-term health-related quality of life^1^. Reported rates of pulmonary complications range from 15 to 50 per cent in recent studies^2–4^, and thus represent a substantial challenge to all clinicians managing patients in the perioperative phase of oesophagectomy. Clinical researchers have focused their efforts on single or bundled interventions in the intraoperative or postoperative setting that may lead to reductions in pulmonary complications^5,6^.

The development of pulmonary complications after oesophagectomy is likely multifactorial and so it is important to consider the strength of evidence behind each intervention along a patient’s pathway. The relative contribution of each intervention to reducing pulmonary complications is unknown. Furthermore, as surgical and anaesthetic interventions are constantly evolving, it becomes increasingly important to identify future areas for research.

This Association of Upper Gastrointestinal Surgery of Great Britain and Ireland (AUGIS) perioperative quality initiative (POQI) aimed to identify clear evidence-based recommendations regarding intraoperative and postoperative care that can reduce pulmonary complications. It also aimed to gain consensus on areas for future research, and on practices that are considered to have no benefit in reducing pulmonary complications. The primary focus of this process was to study potentially modifiable factors that influence postoperative pulmonary complications after oesophagectomy. Preoperative factors affecting development of postoperative pulmonary complications after oesophagectomy were deliberately excluded from the scope of this POQI to avoid duplication with another workstream focused on prehabilitation. The Consensus Statement Group recognized that some of the interventions discussed possibly have an impact on other postoesophagectomy morbidities and outcomes. The group was conscious of this in its discussions, but was clear that the recommendations made here reflect the impact of interventions on pulmonary complications only.

## Methods

This consensus statement represents a collaboration between AUGIS, the Royal College of Surgeons of England Surgical Specialty Leads (RCS SSL) programme (Oesophageal Cancer), and the Perioperative Quality Initiative (POQI). AUGIS and the RCS SSL for Oesophageal Cancer aim to improve the care of patients who undergo oesophagectomy. The POQI is an international multidisciplinary non-profit organization that organizes consensus conferences on clinical topics related to perioperative medicine and surgery^7–11^. Each POQI conference assembles a collaborative group of diverse national or international experts from multiple healthcare disciplines to develop consensus-based recommendations in perioperative medicine or surgery. This partnership combined the expertise of the AUGIS and RCS SSL group with the recognized POQI process.

A modified Delphi method was employed, designed to garner the collective knowledge of this diverse group of experts to answer clinically important questions around perioperative care during oesophagectomy. The clinicians in the POQI consensus meeting were recruited based on their expertise in perioperative management of patients undergoing surgery for oesophageal cancer (AUGIS/POQI Pulmonary Consensus Group Members), and were divided into intraoperative and postoperative research domain groups. Before the conference, topics for discussion at the consensus meeting were longlisted, and a working group of several delegates from each domain group was assembled to systematically review and create a bibliography of relevant literature. This list was used to identify important questions to be addressed in the conference.

Owing to the COVID-19 pandemic, the working groups and POQI conferences were held virtually using an online platform. To ensure focus, all delegates were asked to attend the POQI conferences in full and to block any other commitments. At the first 1-h plenary session of the conference, working groups from the intraoperative and postoperative research groups presented draft consensus statements, and the evidence base on which these had been constructed, to the whole POQI group. The POQI group then split into the intraoperative and postoperative research groups for discussion for 1 h. In the final 1-h plenary session, each working group summarized the breakout discussions and any modifications to the consensus statements to the assembled whole POQI group. At this point, the statements were further refined before voting took place to see whether unanimous consensus could be achieved on each statement presented. After the first conference, the intraoperative and postoperative working groups further refined the statements before a second conference was held, following the same format as the first. In total, two POQI conferences were held and, at the end of the two conferences, POQI statements were either accepted by consensus, or rejected if consensus could not be achieved after discussion and modification.

Groups indicated the strength of evidence underlying practice recommendations using a structure consistent with UK National Institute for Health and Care Excellence guidance.

For publications to be included in this paper, PubMed was searched from 1990 to June 2021 using the following search terms: ‘postoperative pneumonia’, ‘respiratory complication*’, ‘pulmonary complication*’ AND ‘(O)esophagectomy’. The references of relevant articles were reviewed and further articles retrieved if deemed relevant.

## Results

### Consensus statements

#### Surgical approach

**Consensus statement 1**: Either minimally invasive oesophagectomy (MIO) or robot-assisted MIO (RAMIO) is recommended, over open oesophagectomy, to reduce the risk of pulmonary complications. (MIO: Grade B evidence, strong recommendation; RAMIO: Grade D evidence, weak recommendation)

The current evidence base for the use of a minimally invasive approach to oesophagectomy to reduce pulmonary complications is expanding, and minimally invasive techniques are gaining widespread international adoption^12,13^. The current challenge is in distinguishing which specific type of minimally invasive approach confers the maximal benefit in reducing pulmonary complications: laparoscopic abdomen with open chest surgery, open abdomen with thoracoscopic chest surgery, totally minimally invasive oesophagectomy (TMIO), or use of a robotic minimally invasive approach for one or both phases of the procedure. The effects of the different minimally invasive techniques on pulmonary complications have not been directly compared against one another. The POQI group concluded that there is sufficient uncertainty to identify the optimal method for reducing pulmonary complications, and that further trials or registry-based data studies are warranted.

Biere *et al.*^14^ published the TIME trial in 2011, which compared open oesophagectomy with TMIO in 115 patients, with postoperative pulmonary infection as the primary outcome. The results of this multicentre RCT showed a reduction in pulmonary complications from 29 per cent (open group) to 9 per cent (MIO group) (relative risk reduction (RR) 0.30, 95 per cent c.i. 0.12 to 0.76). In 2019, Mariette *et al*.^15^ published the MIRO trial, which randomized 207 patients to either hybrid MIO (laparoscopic abdomen and open chest surgery) or totally open oesophagectomy. They reported a significant reduction in major pulmonary complications associated with hybrid MIO from 30 to 18 per cent (OR 0.50, 95 per cent c.i. 0.26 to 0.96). The most recent single-centre RCT^16^ compared robotic MIO with open oesophagectomy; pulmonary complications were included as a secondary outcome. This reported a significant reduction in pulmonary complications with robotic MIO from 58 to 32 per cent (RR 0.54, 95 per cent c.i. 0.34 to 0.85).

There was considerable debate surrounding this statement and the wording was modified extensively to achieve consensus. The research from RCTs summarized above is overall in favour of a minimally invasive approach to reduce pulmonary complications, but the POQI group had a number of concerns. The TIME trial is based on data from over a decade ago, surgeons were eligible to participate with a minimum of just 10 MIOs, and neither the patients nor the investigators, including those assessing outcomes, were blinded. Nevertheless, it was a multicentre RCT with pulmonary infection as the primary outcome rather than a secondary outcome^17^. In the MIRO trial, the primary outcome was complications with a Clavien–Dindo grade of II or higher, with major pulmonary complications included as a secondary outcome. Although the data for robotic MIO are encouraging, they are from a single-centre RCT and its generalizability has yet to be proven^16^. The ongoing ROBOT-2 multicentre RCT^18^ comparing robotic MIO with standard MIO will provide further information about the efficacy of robotic MIO. The ROMIO study^19^, a UK-based RCT, comparing outcomes in patients receiving hybrid MIO or TMIO with open oesophagectomy recently reported its results, showing no significant differences in outcomes between minimally invasive and open groups. These results were presented after the end of the POQI process but during the manuscript writing period, with full publication still pending. Furthermore, the POQI conferences highlighted discussion regarding the real-world applicability of published RCT data, and particular mention was made of Dutch national registry data that demonstrated an increase in pulmonary complications (OR 1.50, 95 per cent c.i. 1.29 to 1.74) associated with MIO^20^. This suggests that the positive findings in small RCTs might not be reproducible in practice, weakening the strength of the recommendation. However, in the most recent data (published after the consensus meetings) from the International Esodata Study Group, comparing TMIO against hybrid or open oesophagectomy in 8640 patients, TMIO resulted in a lower pneumonia rate and a shorter duration of hospital stay, but at the expense of higher anastomotic leakage rates^18^, supporting the consensus statement.

**Consensus statement 2:** Routine pyloric drainage procedures to reduce pulmonary complications are not recommended. (Grade C evidence, strong recommendation)

Published data assessing the impact of routine pyloric drainage on pulmonary complications are highly heterogeneous and of poor study quality. Arya *et al*.^21^ undertook the most comprehensive review of the literature in 2015, including 25 publications comprising 3172 patients. They studied a range of pyloric interventions including botulinum toxin injection (8.6 per cent), finger fracture (4.2 per cent), pyloroplasty (17.1 per cent), and pyloromyotomy (15.7 per cent). In a pooled analysis from four cohort studies, routine pyloric drainage failed to reduce pulmonary complications (pooled OR 0.77, 95 per cent c.i. 0.46 to 1.28). Other large cohort studies from Tham *et al*.^22^ studying botulinum toxin injection (391 patients), and Antonoff *et al*.^23^ studying pyloromyotomy/plasty or dilatation or dilatation with botulinum toxin (293), failed to show a significant reduction in pneumonia with pyloric drainage procedures. As no high-quality RCT has been conducted to address this specific issue, it is an important research question to be considered for future trials.

#### Perioperative management

**Consensus statement 3:** Targeting normovolaemia to reduce pulmonary complications is recommended. (Grade B evidence, strong recommendation)

Targeting normovolaemia, rather than restricted or liberal fluid administration, has increasingly become the standard approach to intraoperative fluid administration during oesophagectomy. The evidence base for this is good, with two well conducted RCTs, by Mukai *et al*.^24^ (232 patients) and Bahlmann *et al*.^25^ (64), showing marked reductions in pulmonary complications associated with an approach to target normovolaemia. Mukai *et al.*^24^ randomized 232 patients to goal-directed fluid therapy (GDFT) with compound sodium lactate, hydroxyethyl starch, and vasopressors guided by stroke volume variation on Flotrac (Edwards Lifesciences) cardiac output monitors *versus* non-GDFT management, and showed marked reductions in respiratory failure (22.6 *versus* 37.6 per cent; *P* = 0.013), pneumonia (12.2 *versus* 22.2 per cent; *P* = 0.043), and reintubation (3.5 *versus* 16.2 per cent; *P* = 0.001). Despite these RCTs showing the benefits of targeting normovolaemia, there is heterogeneity in results from observational cohort studies, which may represent lack of external validity of the trial findings. However, there was widespread consensus by all POQI experts that targeting normovolaemia is important to reduce pulmonary complications. The techniques used to target normovolaemia and parameters measured remain an important area for future research, as do examining the discrepancy seen in observational data and how best to address this in real-life clinical practice.

**Consensus statement 4:** A lung protective ventilation strategy throughout the operation is recommended. This comprises minimization of peak pressure, limiting tidal volume, optimizing positive end-expiratory pressure, and the use of recruitment manoeuvres to reduce pulmonary complications. (Grade B evidence, strong recommendation)

The evidence base for maintenance of lung protection strategies is good. Michelet *et al*.^26^ undertook an RCT of 52 patients and, with a lung protective strategy only during the one-lung ventilation phase, found improved arterial partial pressure of oxygen (*P*ao_2_)/fraction inspired oxygen (*F*iO_2_) ratios and a reduced duration of postoperative intermittent positive-pressure ventilation of approximately 1 h. In an RCT of 101 patients, Shen *et al*.^27^ showed that low tidal volume during the one-lung ventilation phase was associated with lower rate of pulmonary complications (9.4 *versus* 27.1 per cent; *P* = 0.021). Finally, a systematic review and meta-analysis by Odor *et al*.^5^, which included 16 oesophagogastric RCTs, showed that lung protective ventilation was associated with reduced postoperative pulmonary complications (RR 0.52, 95 per cent c.i. 0.30 to 0.88). Both of the RCTs in patients undergoing oesophagectomy assessed lung protective ventilation during the thoracic or one-lung ventilation phase only, using large tidal volumes (8–9 ml/kg) without positive end-expiratory pressure (PEEP) for the remainder of the operation. Despite this, and on the basis of the study by Odor *et al*.^5^ and current practice, there was widespread agreement with good well conducted published studies to support a lung protective ventilation strategy throughout the operation comprising minimization of peak pressure, limiting tidal volume, optimizing PEEP, and use of recruitment manoeuvres to reduce pulmonary complications.

**Consensus statement 5:** Either thoracic epidural or paravertebral blockade is recommended as the primary method of analgesia to reduce pulmonary complications. (Grade B evidence, strong recommendation)

Thoracic epidurals and paravertebral catheters were considered as the primary method of analgesia among a standard multimodal analgesic approach. The evidence base for both thoracic epidural and paravertebral blockade is good, and the discussion in the POQI expert panel centred around how surgical approach will often determine allocation to one over the other. Several RCTs have compared thoracic epidural with paravertebral blockade, and have failed to show a conclusive difference in pulmonary complications. The most recent Cochrane review, by Yeung *et al*.^28^, included 14 studies and showed no significant difference in respiratory complications between groups (RR 0.62, 95 per cent c.i. 0.25 to 1.52) or pneumonia (RR 0.38, 0.1 to 1.45). It is unlikely that further research in this area will change this recommendation; however, it was acknowledged that there is an ongoing RCT^29^ evaluating this issue specifically in the context of MIO.

#### Oral intake strategies and delayed gastric emptying

**Consensus statement 6:** Routine use of nasogastric tubes to reduce the risk of pulmonary complications is not recommended (Grade D evidence, weak recommendation)

Delayed gastric emptying (DGE) is a multifactorial and poorly understood process that can result in retention of secretions and oral intake in the gastric conduit, risking acute conduit dilatation and aspiration. The lack of consensus on the prevention, management, and even the precise definition of DGE is a recognized problem. One recent Delphi consensus^30^ attempted to address this, defining postoperative DGE as a daily nasogastric tube output exceeding 500 ml between days 5 and 14. However, although nasogastric tubes are commonly used after oesophagectomy to decompress the conduit, they are rarely kept in place for long enough to aid in diagnosis according to this definition. Recent evidence in colorectal and bariatric surgery has resulted in the removal of nasogastric tube use from enhanced recovery protocols on the basis that they do not aid return of gut function and may actually represent an aspiration risk^31,32^.

Despite traditional surgical dogma mandating nasogastric tube use after oesophagectomy, there is little supporting evidence. A meta-analysis^33^ of seven studies, including four RCTs, found that early removal or omission of nasogastric tubes after oesophagectomy did not result in any difference in mortality or respiratory complications compared with nasogastric tube use. Since this meta-analysis, a further small RCT^34^ (80 patients) has shown no adverse outcomes in terms of rates of pneumonia, anastomotic leak, and nasogastric tube reinsertion in comparisons of removal on postoperative day (POD) 1 or 7. The available evidence is likely to be underpowered, explaining the weak recommendation and the need for further research.

**Consensus statement 7:** Commencement of clear oral fluids in the immediate postoperative phase is recommended as this does not increase the risk of pulmonary complications. (Grade C evidence, strong recommendation)

A 2016 meta-analysis^35^ compared early with late oral feeding. Groups were defined by individual study authors; early feeding ranged from oral fluids on POD 1–3, and late feeding on POD 3–6. RCT data demonstrated no significant difference in the risk of pneumonia; however, when cohort data were pooled together with those from RCT reports, a significantly lower risk of pneumonia was observed in the early-fed compared with the late-fed group (OR 0.60, 95 per cent c.i. 0.41 to 0.89; *P* = 0.01). A further recent RCT^36^ (132 patients) randomized patients to immediate oral feeding or feeding after POD 5. The early oral diet consisted of 250 ml water on the day of surgery and 500 ml liquid oral diet on day 1, which was increased gradually thereafter. Although 38 per cent of patients in the early feeding group deviated from the treatment pathway owing to complications or inability to progress, pneumonia rates did not differ significantly between groups and the study concluded that direct oral feeding is safe and feasible.

#### Route and formulation of postoperative feeding

**Consensus statement 8:** Enteral feeding is recommended in preference to parenteral nutrition, to reduce the risk of pulmonary complications. (Grade C evidence, strong recommendation)

Variation in practice during the postoperative phase exists with reference to the type of nutrition, the delivery route, and its timing. Nutritional supplementation can be given via multiple enteral routes including nasojejunal or jejunostomy feeding tubes, as well as orally, or it can be administered parenterally.

Peng *et al*.^37^ reported the results of a meta-analysis that compared enteral with parenteral nutrition after oesophagectomy. The majority of these RCTs were small; all were carried out in Japanese or Chinese populations and used nasojejunal (7 studies), jejunostomy (1) or nasoduodenal feeding (2) routes to feed patients in the enteral arms; no trials used early oral nutrition. In this analysis, enteral nutrition was associated with fewer pulmonary complications. Comparing individual feeding routes, one UK-based RCT^38^ (121 patients) compared early jejunostomy feeding with delayed oral intake (after contrast swallow typically 7–10 days after surgery) in oesophagectomy, total gastrectomy, and pancreatectomy. Rates of overall complications (16, 32.8 per cent *versus* 7, 50.9 per cent; *P* = 0.044), and specifically the incidence of chest infections (5, 7.8 per cent *versus* 12, 21.1 per cent; *P* = 0.036), were significantly lower in the early jejunostomy feeding group, although the data were variable. A small cohort study^39^ and an RCT^40^ comparing jejunostomy with nasojejunal feeding both reported slightly lower pulmonary complication rates with jejunostomy use: 17 *versus* 22.2 per cent (*P* = 0.037)^39^ and 34 *versus* 41 per cent (*P* not stated)^40^. Conversely, a recent meta-analysis^41^ comparing jejunostomy use with non-jejunostomy-based feeding (predominantly nasojejunal or oral, but also some parenteral nutrition) reported lower rates of pulmonary complications in the non-jejunostomy group. On the balance of evidence for its safety, combined with the physiologically preferential nature of enteral feeding, a strong recommendation for feeding via the enteral route was made, although no specification of type (jejunostomy, transnasal, or oral) could be given.

**Consensus statement 9:** There is no evidence that specific nutritional formulae reduce the incidence of pulmonary complications. (Grade D evidence, no recommendation)

The potential for specific feed formulations to improve outcomes has been a recent subject of great interest. Immunonutrition comprises the supply of specific nutrients in an attempt to modify inflammatory or immune responses. It has been theorized that this could improve immune function after surgery, which may reduce the risk of complications. However, evidence regarding postoperative use of immunonutrition is limited to a few small studies^42–44^, which failed to demonstrate any difference between immunonutrition and standard formulations, with no evidence specific to oesophagectomy. No recommendation could be made.

#### Physiotherapy and early mobilization

**Consensus statement 10:** Chest physiotherapy and early mobilization are recommended to reduce pulmonary complications. (Grade C evidence, strong recommendation)

Early mobilization and routine physiotherapy are commonly encouraged after oesophagectomy, and are keystones of most enhanced recovery protocols aiming to improve recovery and reduce morbidity. However, evidence for the impact of specific physiotherapeutic interventions on respiratory complications after oesophagectomy is less clear.

Only two studies^45,46^ have specifically examined the impact of respiratory physiotherapy on complications after oesophagectomy. They found significant benefit from respiratory physiotherapy, although both were small retrospective studies of poor quality. A 2006 systematic review^47^ of 13 RCTs of respiratory physiotherapy in abdominal surgery concluded that physiotherapy provided no benefit. A more recent 2020 meta-analysis^5^ of respiratory physiotherapy in abdominal and thoracic surgery reported a significantly reduced risk of respiratory complications (RR 0.55, 95 per cent c.i. 0.32 to 0.93); however, this was disproportionately weighted by a single large trial reporting significant benefit, whereas 11 others showed no significant difference.

Early postoperative mobilization is advocated after oesophagectomy to improve lung volumes, and reduce postoperative atelectasis and morbidity. However, evidence to support early mobilization as a stand-alone intervention in this population is limited and of low quality. One small retrospective study (118 patients) published by Hanada *et al*.^48^ in 2018 specifically assessed this; a regression analysis showed that early mobilization reduced postoperative atelectasis in patients who had undergone MIO (OR 1.87, 95 per cent c.i. 1.36 to 2.57; *P* < 0.001)

Incentive spirometry is a cost-effective adjunct that is often prescribed to patients in the postoperative environment. Despite this, there is an absence of evidence supporting its efficacy in patients who have undergone oesophagectomy and data from other surgical populations is unconvincing. A 2014 Cochrane review by Nascimento *et al*.^49^ assessed the impact of incentive spirometry on respiratory complications in comparison to other breathing exercises and no breathing exercises in upper abdominal surgery, concluding that there was no benefit from the intervention (RR 0.83, 95 per cent c.i. 0.51 to 1.34). Many of the reviewed studies lacked methodological rigour and had a high risk of bias. Despite the modest quality of evidence, there was consensus for a strong recommendation for physiotherapy and mobilization to reduce complications. Further high-quality research is required to investigate the effect of specific interventions, such as incentive spirometry, on outcomes.

#### Chest drains

**Consensus statement 11:** More than one thoracic drain to reduce pulmonary complications is not recommended. (Grade C evidence, weak recommendation)

Chest drains are placed almost universally after oesophagectomy to avoid pleural effusions or persistent pneumothorax, but they may vary in number, type, placement (unilateral *versus* bilateral, intercostal *versus* transhiatal), and use (free drainage *versus* negative pressure, variable criteria for drain removal). Furthermore, drains can be a significant source of discomfort and therefore a potential risk factor for respiratory complications.

Comparing the use of single *versus* two chest drains after oesophagectomy, retrospective studies^50–52^ have suggested equivalence in terms of respiratory complications, but a reduction in pain scores with fewer drains^50,51^. Similarly, transhiatal drain placement may result in lower pain scores and analgesia use^52–54^, without any significant differences in respiratory or other complications.

Two studies^55,56^ assessing high (250 or 300 ml) *versus* low (50 or 150 ml) daily drainage volume thresholds for drain removal found no difference in outcomes. Finally, one RCT^57^ has assessed the impact of negative pressure (−15 mmHg) *versus* free drainage for chest drains after oesophagectomy, with no difference in rates of pneumothorax or hydrothorax. A recent systematic review^58^ has summarized the sparse evidence for chest drain use, but suggests that fewer drains and more permissive removal strategies may be employed without negatively influencing outcomes and may result in reduced patient discomfort.

#### Enhanced recovery after surgery

**Consensus statement 12:** Enhanced recovery pathways are recommended to reduce pulmonary complications. (Grade C evidence, strong recommendation)

The use of standardized clinical care pathways after surgery is increasingly being advocated across specialties. Enhanced recovery after surgery (ERAS) protocols include multiple processes across the entire of care pathway; variable levels of evidence mean that there is no firm consensus on what should be included in individual protocols^29^. Rather, the aim is to ensure standardized, evidence-based care across systems, to accelerate and improve recovery.

Although several systematic reviews and meta-analyses have been published examining the effect of ERAS on outcomes after oesophagectomy, these are based on small studies (fewer than 200 participant) of moderate quality. Several small RCTs^59–62^ comparing ERAS with conventional care have been conducted, all based on single-centre Chinese populations, and have been included in several recent meta-analyses^63,64^. Although the included studies vary significantly between these meta-analyses (owing to differing inclusion criteria), their reported findings are similar. In 2020, Huang *et al*.^64^ pooled findings from five studies (646 patients), reporting a reduced risk of pulmonary complications for ERAS care compared with conventional care (RR 0.42, 95 per cent c.i. 0.21 to 0.82). The 2020 analysis by Triantafyllou *et al*.^63^ included seven studies (1017 patients) (OR 0.45, 95 per cent c.i. 0.31 to 0.65). In 2015, Markar *et al*.^65^ considered four studies (638 patients) and similarly confirmed a reduction in pulmonary complications in the ERAS group (OR 0.52, 95 per cent c.i. 0.36 to 0.77; *P* = 0.001).

The precise components of the ideal ERAS pathway are a topic of debate. The multifactorial nature of ERAS programmes makes assessing the impact of individual component interventions difficult, if not impossible. It has been suggested that the standardization of care itself, rather than the nature of the actual care given, may be an important factor in the success of ERAS pathways. Regardless, evidence in oesophagectomy and other surgical fields is unequivocal in supporting the beneficial effects of ERAS, and these continue to be adopted at pace.

### Research recommendations

During formulation of the consensus statements, there were clear and obvious areas where the expert panel felt that more research was required:

• What is the optimal minimally invasive surgical technique (for example, hybrid MIO, TMIO or RAMIO) to reduce pulmonary complications?
• Do pyloric drainage procedures reduce pulmonary complications?
• What are the benefits or harms of nasogastric tube use after oesophageal surgery?
• What is the optimal manner of progressing oral intake after oesophageal surgery?
• What is the optimal route and timing of enteral nutrition to reduce pulmonary complications?
• Does incentive spirometry in the postoperative setting reduce pulmonary complications?
• What is the optimal method of pleural drainage to reduce pulmonary complications?

## Additional recommendations for research

There have been numerous studies on multiple facets of the care of patients undergoing oesophagectomy. Most of these assessed pulmonary complications as a secondary outcome or assessed markers of lung injury without clinical effects. Many frequently performed interventions have received minimal research attention. The following questions were identified either through literature reviews or expertise of the consensus group participants as areas of interest; current practice and evidence were insufficient to merit full discussion and consensus recommendation, but they were agreed as research priorities on the topic of reducing pulmonary complications after oesophagectomy.

*Additional research question 1: What is the optimal position during the thoracic portion of minimally invasive oesophagectomy to reduce pulmonary complications?*

Two retrospective Japanese cohort studies^66,67^ including 319 patients have compared the effects of the prone *versus* lateral decubitus position on the incidence of pulmonary complications after thoracoscopic oesophagectomy. Pulmonary complications were secondary outcomes, defined as pneumonia on imaging/requiring antibiotics, atelectasis on imaging, or a fever over 38°C. Both studies reported a lower incidence of pulmonary complications in the prone position compared with the lateral position (15.4 *versus* 30.8 per cent, *P* < 0.05^67^; 7 *versus* 30 per cent, *P* < 0.01^66^). The POQI expert group concluded that, although surgical positioning is largely dependent on the surgical technique and operator familiarity, the topic is worthy of further research as pulmonary complications were poorly defined secondary outcomes and positioning could be changed to reduce the incidence.

*Additional research question 2: Does continuous positive airway pressure to the deflated lung during one-lung ventilation reduce pulmonary complications?*

A recent small RCT^68^ compared the effects of a continuous positive airway pressure (CPAP) of 5 cmH_2_O applied to the deflated lung in 30 patients undergoing robot-assisted thoracolaparoscopic oesophagectomy. The study showed reduced markers of lung inflammation (interleukin (IL) 1α, IL-1β, IL-8, IL-10, tumour necrosis factor α, macrophage inflammatory protein 1α, pulmonary and activation-regulated chemokine) in patients receiving CPAP (*P* < 0.05) but this did not translate into reduced pneumonia (RR 0.8, 95 per cent c.i. 0.27 to 2.41; *P* = 0.69). There are plausible mechanisms by which CPAP to the deflated lung may reduce pulmonary complications, and the effect on clinical outcomes may be seen to a greater extent in patients undergoing open surgery. Further research assessing different levels of intraoperative CPAP to the deflated lung, a wider range of pulmonary complications, and in a wider variety of surgical techniques would be beneficial.

*Additional research question 3: Do perioperative inhaled long-acting β-2-adrenoreceptor agonists reduce pulmonary complications?*

A single UK multicentre placebo controlled RCT^69^ evaluated the effect of salmeterol on postoperative pulmonary complications in patients undergoing oesophagectomy using a variety of operative techniques. They reported a lower incidence of pneumonia (OR 0.39, 95 per cent c.i. 0.16 to 0.96), and lower levels of ICAM-1 and soluble receptor for advanced glycation end-products (both markers of endothelial damage) in the salmeterol group, but no difference in the incidence of acute lung injury (ALI) (OR 1.25, 0.71 to 2.22). However, pneumonia was not clearly defined and was only a secondary outcome. A systematic review and meta-analysis by Odor *et al.*^5^, including 16 oesophagogastric RCTs, showed that prophylactic inhaled β-agonists were not associated with a reduction in pulmonary complications in 405 patients (RR 0.93, 95 per cent c.i. 0.67 to 1.29; *P* = 0.65) or respiratory infections (RR 0.62, 0.31 to 1.24; *P* = 0.18). Salmeterol is cheap, simple to administer, and has a low side-effect profile, so further studies evaluating its efficacy in the oesophagectomy population were considered worthwhile by the POQI expert group.

*Additional research question 4: Do non-steroidal anti-inflammatory drugs reduce the risk of pulmonary complications when used as part of a multimodal analgesic package?*

The effect of non-steroidal anti-inflammatory drugs (NSAIDs), when used as part of a multimodal analgesia technique in an ERAS programme, can help optimize analgesia^70,71^. However, there is no literature on their effect on pulmonary complications. Optimal analgesia is widely considered to be key in preventing postoperative respiratory complications and facilitating return to function. On this basis, the POQI group concluded that there is a rationale for investigating the effect of NSAIDs on pulmonary complications, most likely as part of a bundle of measures.

*Additional research question 5: Do neutrophil elastase inhibitors reduce pulmonary complications?*

The effect of the selective neutrophil elastase inhibitor sivelestat on pulmonary complications after oesophagectomy was described in a systematic review and meta-analysis of 13 studies^72^. Sivelestat lowered the incidence of ALI (RR 0.27, 95 per cent c.i. 0.08 to 0.93) and reduced the duration of postoperative mechanical ventilation (standardized mean difference −1.41, −2.63 to −0.19), but did not affect the incidence of pneumonia (RR 0.84, 0.47 to 1.50; *P* = 0.86). The included studies were all conducted in Japan and tended to have fewer than 20 patients in each arm; 8 were non-randomized and only 1 of the randomized studies described clear blinding and allocation concealment. Sivelestat was unknown to the POQI panel and is not licensed for use in the UK. Eli Lilly announced in 2020 that it would be developing the drug further in phase II trials for respiratory failure in the USA. Further large well conducted randomized studies in the oesophagectomy population might be worthwhile.

*Additional research question 6: Does total intravenous anaesthesia reduce pulmonary complications compared with volatile anaesthetics?*

Two retrospective studies^73,74^ compared outcomes after oesophagectomy in patients receiving total intravenous anaesthesia (TIVA) or volatile anaesthesia. The primary outcome in the study by Jun *et al*.^73^ was recurrence-free survival after volatile or propofol anaesthesia for oesophagectomy. Pulmonary complications, defined using European Perioperative Clinical Outcome definitions, were recorded alongside many other organ injuries. There was no difference between groups in duration of postoperative mechanical ventilation exceeding 48 h (5.2 *versus* 6.4 per cent; *P* = 0.659), pneumonia (15.7 *versus* 15.0 per cent; *P* = 0.910) or ALI/acute respiratory distress syndrome (2.6 *versus* 3.1 per cent; *P* = 0.817). The incidence of pneumonia was low in both groups in this study. Zhang and Wang^74^ compared the effects of propofol TIVA with sevoflurane in terms of the risk of developing postoperative pneumonia in a retrospective cohort study of 1659 patients, of whom 78 had TIVA. Before and after propensity matching, there were no differences in postoperative pneumonia (sevoflurane 7.7 per cent *versus* TIVA 6.4 per cent; *P* = 0.754) or reintubation (2.6 *versus* 0 per cent respectively; *P* = 0.155). As use of TIVA becomes increasingly mainstream in cancer surgery, further prospective research in larger study populations or registry data assessing a wider variety of pulmonary complications is worthwhile.

*Additional research question 7: Does intraoperative bronchoscopic targeted therapy reduce pulmonary complications?*

Intraoperative fibreoptic bronchoscopy and targeted aspiration of respiratory secretions is commonly performed during oesophagectomy as part of the anaesthetic technique for one-lung ventilation. There is heterogeneity in whether and how the technique is performed, but it is usual for the trachea and main bronchi of the deflated lung to be suctioned before reinflation, and/or for the ventilated lung to be inspected and suctioned after two-lung ventilation has been restarted. The literature review group could not find any studies referring to bronchoscopic targeted therapy on pulmonary complications, and there was variation in practice among the anaesthetists in the POQI group. Research addressing the value of this practice in reducing pulmonary complications and the optimal method is warranted.

*Additional research question 8: What is the optimal ventilation strategy (one- or two-lung ventilation) during minimally invasive oesophagectomy to reduce pulmonary complications?*

Both one- and two-lung ventilation can be used during the thoracoscopic component of MIO. Current use is dependent on the surgical technique, and probably institutional preference. Two trials^75,76^ including 133 patients have compared the effects of two- *versus* one-lung ventilation on parameters including intraoperative *P*ao_2_/*F*iO_2_ ratios, intraoperative arterial partial pressure of carbon dioxide, intraoperative airway pressures, postoperative C-reactive protein, reintubation rates, and non-specified respiratory complications. There were small predictable differences in intraoperative ventilation parameters, but no difference in pulmonary complications (23.9 *versus* 16.7 per cent for one- *versus* two-lung ventilation; *P* = 0.37). The POQI group concluded that the choice of ventilation technique will depend on the surgical technique. Further work evaluating the effects on postoperative pulmonary complications would be worthwhile.

### Strengths and limitations

The POQI group has used a well established methodology to combine a literature review with expert interpretation and opinion to produce pragmatic consensus statements on areas in which the optimal approach is unclear. Although care was taken to select a diverse group of experts, this remains a discussion between a limited sample of clinicians. No formal systematic review or meta-analysis was included; this was to keep the methodology pragmatic. Any uncertainty or persisting discord has been highlighted in the text accompanying each statement. Some statements generated more discussion than others, which is likely a reflection of the ambiguity of the available evidence and also the acceptability of any proposed modifications to practice. Although this can be seen as a limitation, in pragmatic terms this discussion is a valuable insight into the likelihood of the adoption of any proposed recommendations.

## Conclusions

Pulmonary complications after oesophagectomy are a significant challenge for patients and perioperative clinicians. This POQI working group has developed evidence-based consensus recommendations on a number of intraoperative and postoperative interventions to reduce pulmonary complications. However, there remain significant areas where the evidence base is weak and could be improved significantly. In addition to identifying evidence-based recommendations, the working group has highlighted key topics that funders and researchers should focus on to continue the quality improvement drive for pulmonary complications after oesophagectomy.

## Collaborators

AUGIS/POQI Pulmonary Consensus Group: M. Mythen, N. Maynard, M. Grocott, T. Underwood, O. Pickering, P. Singh, S. Markar, D. Levett, C. Alan, N. Tewari, F. Noble, K. Moorthy, M. Oliver, S. Chidambaram, A. Wickham, J. Gossage, P. Pucher, A. Bull, J. Sultan, F. Huddy, S. Davies, J. Weblin, M. West

## AUGIS/POQI Pulmonary Consensus Group Members

- Faculty
  - Monty Mythen – Consultant in Intensive Care Medicine, University College Hospital NHS Foundation Trust
  - Nick Maynard – Consultant OG Surgeon, Oxford University Hospitals NHS Foundation Trust
  - Mike Grocott – Consultant in Intensive Care Medicine, University Hospital Southampton NHS Foundation Trust
  - Tim Underwood – Consultant OG Surgeon, School of Cancer Sciences, University of Southampton
- Consensus meeting coordination
  - Oliver Pickering – Clinical Research Fellow, School of Cancer Sciences, University of Southampton
- Intraoperative Interventions Group:
  - Pritam Singh – Consultant OG Surgeon, Royal Surrey NHS Foundation Trust
  - Sheraz Markar – OG Surgeon, Oxford University Hospitals NHS Foundation Trust. Assistant Professor, Department of Molecular Medicine & Surgery, Karolinska Institutet, Sweden
  - Oxford University Hospitals NHS Foundation Trust
  - Denny Levett – Consultant in Intensive Care Medicine, University Hospital Southampton NHS Foundation Trust
  - Charlotte Alan – Consultant Anaesthetist, University Hospital Southampton NHS Foundation Trust
  - Nilanjana Tewari – Consultant OG Surgeon, University Hospitals Coventry and Warwickshire NHS Trust
  - Fergus Noble – Consultant OG Surgeon, University Hospital Southampton NHS Foundation Trust
  - Krishna Moorthy – Consultant OG Surgeon, Imperial College Healthcare NHS Trust
  - Matt Oliver- Consultant Anaesthetist, University College Hospital NHS Foundation Trust
  - Swathikan Chidambaram – Surgical Trainee, Imperial College Healthcare NHS Trust
  - Alex Wickham – Consultant Anaesthetist, Imperial College Healthcare NHS Trust
- Postoperative Interventions Group:
  - James Gossage – Consultant OG Surgeon, Guy’s and St Thomas’ NHS Foundation Trust
  - Phillip H Pucher – Consultant OG Surgeon, Portsmouth Hospitals University NHS Trust
  - Alexander Bull – Clinical Research Fellow, Guy’s and St Thomas’ NHS Foundation Trust
  - Javed Sultan – Consultant OG Surgeon, Salford Royal Hospital, Northern Care Alliance NHS Foundation Trust. Honorary Senior Lecturer, Division of Cancer Sciences, School of Medical Sciences, University of Manchester
  - Fiona Huddy – Specialist OG Dietitian, Royal Surrey NHS Foundation Trust
  - Sarah Davies – Highly Specialist Dietitian UGI Cancers, University Hospital Southampton/ Gloucestershire Hospitals NHS Foundation Trust
  - Jonathan Weblin – Physiotherapist, University Hospitals Birmingham NHS Foundation Trust
  - Malcolm A. West – Consultant Colorectal and Complex Cancer Surgeon, University Hospital Southampton NHS Foundation Trust. Associate Professor of Colorectal Surgery, School of Cancer Sciences, Faculty of Medicine, University of Southampton.

## Supplementary Material

znac193_Supplementary_Data
