# Supplementary material for: Association of Upper Gastrointestinal Surgery of Great Britain and Ireland (AUGIS)/Perioperative Quality Initiative (POQI) consensus statement on intraoperative and postoperative interventions to reduce pulmonary complications after oesophagectomy
Source: Br J Surg. 2022 Aug 24;109(11):1096–106. doi: 10.1093/bjs/znac193 (PMC10364741; doi:10.1093/bjs/znac193)
Supplement: znac193_Supplementary_Data [file znac193_supplementary_data.docx]

**Supplementary Tables**

Table S1. We recommend either MIE or RAMIE, over open oesophagectomy, to reduce the risk of pulmonary complications (MIE Grade B strong recommendation; RAMIE Grade D evidence, weak recommendation).

| **Study** | **Study design** | **Origin and year** | **Sample size** | **Intervention** | **Outcome measures of pulmonary complications** |
| --- | --- | --- | --- | --- | --- |
| Biere et al | Multicentre, open-label RCT | 2012  Netherlands, Italy, Spain | 115 | 1. Open Oesophagectomy    2. MIE | 1. Postoperative Pulmonary infection |
| Van der Sluis et al | Single centre RCT | 2019  Netherlands | 112 | 1. Open Oesophagectomy    2. Robot Assisted MIE (RAMIE) | 1. CD 2 or greater complication    2. Pulmonary complications: (pneumonia, pneumothorax, PE, ARDS) |
| Mariette et al | Multicentre, open-label RCT | 2019  France | 207 | 1. Open Oesophagectomy  2. Hybrid MIE | 1. CD Grade 2 or greater complication  2. Pulmonary complications. |

MIE; Minimally-invasive oesophagectomy, CD; Clavian- Dindo, PE; Pulmonary embolus, ARDS; Acute respiratory distress syndrome.

Table S2: We do not recommend routine pyloric drainage procedures to reduce pulmonary complications. (Grade C evidence; strong recommendation).

| **Study** | **Study design** | **Origin and year** | **Sample size** | **Intervention** | **Outcome measures of pulmonary complications** |
| --- | --- | --- | --- | --- | --- |
| Arya et al | Systematic review | 2015,  UK | 25 papers; 3172 patients | Pyloric management strategies:  no intervention, botulinum toxin injection, finger fracture, pyloroplasty, and  pyloromyotomy | Pulmonary complications,    Hospital mortality  Anastomotic Leak  Delayed Gastric Emptying (DGE)  Bile reflux |
| Tham et al | Feasibility study (prospective) | 2018  UK | 391 patients | 1. Intraoperative pyloric botulinum toxin injections (228)    2. No Botox injections (163) | Primary outcome:  DGE - No direct comparison of botox injections and incidence of pneumonia. |
| Nevo et al | Retrospective database study | 2020,  Canada | 94 | 1.Endoscopic pyloromyotomy (52)  2.Surgical pyloromyotomy (42) | Pulmonary complications |
| Eldaif et al | Retrospective database study | 2014, USA | 322 | 1.Botox (78)  2.Pyloromyotomy (45)  3. Pyloroplasty (199) | Pneumonia |
| Antonoff et al | Retrospective database study | 2014,  USA | 293 | 1. No drainage (44)  2. Pyloromyotomy/ pyloroplasty  3. Dilation  4. Dilation + Botox | Aspiration pneumonia |

DGE: Delayed gastric emptying

Table S3. We recommend targeting normovolaemia to reduce pulmonary complications. (Grade B evidence; recommendation strong).

| **Study** | **Study design** | **Origin and year** | **Sample size** | **Intervention** | **Outcome measures of pulmonary complications** |
| --- | --- | --- | --- | --- | --- |
| Mukai et al | Multicentre RCT | 2020,  Japan | 232 | GDT (SVV) Flotrac, CSL & HES, pressors  Non GDT | 1. Mortality  2. Reoperation for bleeding  3. Anastomotic leakage  4. Pneumonia  5. Reintubation  6. >48 h ventilation |
| Haas et al | Prospective observational | 2012,  Germany | 27 | 1. GDT | 1. Extravascular Lung Water Index  2. Operative time and blood loss  3. Inotrope requirements  4. Diuresis requirements  5. Crystalloids and colloid requirements |
| Bahlmann et al | RCT | 2015,  Sweden | 64 (59 in analysis) | GDT (SVV) with FloTrac, colloid, dobutamine/ NA | 1. POD5 and 30 complications  2. LOS in hospital and ICU  3.Time needed for normal bowel function  4. Mortality  5. Inotrope use  6. Operative time and blood loss |
| Oya et al | Single centre, prospective, observational | 2019,  Japan | 24 | Bioelectrical impedance analysis | 1. Body weights and volume measurements (Extra Cellular Water, Total Body Water, Internal Cellular Water, Fat Free Mass)  2. Infective complications (infiltrate on CXR/ CT, purulent sputum, rise in WCC or CRP) |
| Hikasa et al | Single-centre retrospective cohort | 2020,  Japan | 135 | MIE, compared pts with fluid balance above or below median | Complications –CD grade >2  -Arrythmias  -Thrombosis  -AKI needing haemodialysis  -Pneumonia (on imaging, Abx, mechanical ventilation) |

Abx; Antibiotics, AKI; Acute kidney injury, CD; Clavian-Dindo, CXR; Chest X-ray, GDT; Goal directed therapy, LOS; Length of stay, MIE; Minimally invasive oesophagectomy, POD; Postoperative Day, RCT; Randomised controlled trial, SVV; Stroke volume variation.

Table S4. We recommend a lung protective ventilation strategy throughout the operation. This comprises minimisation of peak pressure, limiting tidal volume, optimising PEEP and the use of recruitment manoeuvres to reduce pulmonary complications (Grade B evidence; strong recommendation).

| **Study** | **Study design** | **Origin and year** | **Sample size** | **Intervention** | **Outcome measures of pulmonary complications** |
| --- | --- | --- | --- | --- | --- |
| Michelet et al. | RCT | 2006, France | 52 | Conventional ventilation (throughout VT 9ml/kg & no PEEP)  Protective (VT 9ml/kg in TLV 🡪 5ml/kg in OLV, PEEP 5) | 1. Inflammatory cytokines  2. PaO_2_/FiO_2_ ratio, EVLWI |
| Shen et al. | RCT | 2013, China | 101 | Low VT (5 mL/kg & 5 cm PEEP)  Conventional: 8ml/kg CMV | 1. Inflammatory cytokine levels  2. Pulmonary complications |
| Odor et al | MA & SR | 2020, UK | 117 studies, 21940 pts  16 UGI RCTs | Multiple considered:  ERAS  Prophylactic mucolytic  Lung protective ventilation  Restrictive v liberal fluids  Epidural analgesia  Goal directed haemodynamic therapy  Prophylactic inhaled beta agonist  Incentive spirometry  High intraoperative FiO2 (0.8) | European Perioperative Clinical Outcome  consensus statement |

EVLWI; Extravascular lung water index, FiO_2_; Fraction of inspired oxygen, MA; Meta-analysis, OLV; One-lung ventilation, PaO_2_; Partial pressure of oxygen, PEEP; Positive end-expiratory pressure, RCT; Randomised controlled trial, SR; Systematic review, TLV; Two-lung ventilation, VT; Tidal volume.

Table S5. We recommend either thoracic epidural or paravertebral blockade as the primary method of analgesia to reduce pulmonary complications. (Grade B evidence; strong recommendation).

| **Study** | **Study design** | **Origin and year** | **Sample size** | **Intervention** | **Outcome measures of pulmonary complications** |
| --- | --- | --- | --- | --- | --- |
| Kingma et al | Protocol for RCT | 2020, Netherlands | 192 | Thoracic epidural block  Paravertebral block | Ongoing study |
| Yeung et al | Cochrane review | 2016 | 698 |  | No significant difference in respiratory complications: 5 studies, 280 participants. RR 0.62 (95%CI 0.26-1.52), p = 0.30  Pneumonia: favours TEA but RR 0.38, 95%CI 0.1-1.45, p = 0.16 |
| Bimston et al | Prospective, randomized study | 1999,  USA | 50 |  | No significant difference between FEV1 and FVC on POD 0, 1, 2, 3  No significant difference in pulmonary complications (as defined below)   - Pneumonia: CXR findings, fever, raised WCC, positive sputum culture - Atelectasis/mucus plugging/parenchymal collapse requiring bronchoscopy - Prolonged air leak: >72 hour after operation |
| Casati | RCT | 2006, Italy | 42 |  | PaO_2_/FiO_2_ ratio reduced in both groups post-op |
| De Cosmo | RCT | 2002, Italy | 50 |  | Incidence of reintubation: 4 in both groups  Incidence of respiratory distress: 1 in TEA vs 2 in PVB  Incentive spirometry (ability to achieve spirometry volume >2L) achieved by 87% in TEA vs 78% in PVB |
| Grider | Double blinded RCT | 2012 | 75 |  | Incentive spirometry data (ability to achieve spirometry volume >2L): 87% in TEA with opioid vs 78% in PVB vs 55% in epidural LA only  2 patients in TEA group were reintubated |
| Kaufmann | 1x centre retrospective cohort | 2019, Germany | 335 | Thoracic epidural block | PPC defined by International Consensus on Standardization of Data Collection for Complications Associated with Oesophagectomy.   1. Transfusion of PRBCs: OR 1.9 (95%CI 1.2–3 95) for PPCs 2. Absence of TEA: OR 2.0 (95%CI 1.01–3.8) |

CXR; Chest x-ray, FEV1; Forced expiratory volume, FVC; Forced vital capacity, PPC; Post-operative pulmonary complication, PRBC; Packed red blood cells, PVB; Paravertebral block, RCT; Randomised controlled trial, TEA; Thoracic epidural anaesthesia, WCC; White cell count.

Table S6: We do not recommend the routine use of NGTs to reduce the risk of pulmonary complications. (Grade D evidence; weak recommendation)

| **Author** | **Study design** | **Origin and year** | **Sample size** | **Intervention** | **Outcome measures of pulmonary complications** |
| --- | --- | --- | --- | --- | --- |
| Konradsson et al | Consensus statement | International | N/a | International Delphi process to define early and late DGE, as well as a late symptom grading tool. | Early: Greater than 500ml diurnal NGT measured on the morning of postoperative day 5 or later > 100% increased gastric tube width on frontal CXR projection, with air-fluid level. |
| Gustafsson et al | ERAS society guidelines for elective colorectal surgery | International | N/a | Use of NGT during elective colorectal surgery | Postoperative nasogastric tubes should not be used routinely; if inserted during surgery, they should be removed before reversal of anaesthesia. |
| Thorell et al | ERAS society guidelines for bariatric surgery | International | N/a | Use of NGT during bariatric surgery | Routine use of nasogastric tube is not recommended postoperatively |
| Weijs et al | Meta-analysis of seven studies | The Netherlands, Sweden & Japan | n = 608 | Perioperative compared to routine (delayed) NGT removal | Not reported in 4 studies, only infectious complications in one, symptoms and CXR in one and positive sputum cultures in final study. |
| Hayashi et al | RCT | Japan | n=80 | NGT removed on POD 1 or POD7 | Post-op pneumonia – body temp > 38 C or raised white cell count or C-reactive protein and infiltrative shadow seen on CXR. |

CXR; Chest x-ray, DGE; Delayed gastric emptying, ERAS; Enhanced recovery after surgery, NGT; Nasogastric tube, POD; Postoperative day, RCT; Randomised controlled trial.

Table S7: We recommend commencement of clear oral fluids in the immediate postoperative period as this does not increase the risk of pulmonary complications. (Grade C evidence; strong recommendation).

| **Author** | **Study design** | **Origin and year** | **Sample size** | **Intervention** | **Outcome measures of pulmonary complications** |
| --- | --- | --- | --- | --- | --- |
| Willcutts et al | Meta-analysis | 2016, international | 8 studies (n not given) | Risk of pneumonia in early feeding when compared to late feeding in UGI surgery. | Postoperative pneumonia definitions not given |
| Berkelmens et al | Open-label RCT | 2020, The Netherlands, Sweden | n=148 | Intervention group; Day 0, sips up to 250ml, day 1, 500ml of liquid oral intake, increased 250ml per day till day 5 vs the control group starting this on day 5 with jejunostomy feeding in the interim. | Pulmonary complications were defined as pneumonia scored using the Uniform Pneumonia Score; aspiration pneumonia, defined as pneumonia following aspiration of saliva, liquid or solid food or vomit; acute respiratory distress syndrome, defined as respiratory insufficiency requiring treatment |

RCT; Randomised controlled trial.

Table S8: We recommend enteral feeding in preference to parenteral nutrition, to reduce the risk of pulmonary complications. (Grade C evidence; strong recommendation).

| **Author** | **Study design** | **Origin and year** | **Sample size** | **Intervention** | **Outcome measures of pulmonary complications** |
| --- | --- | --- | --- | --- | --- |
| Peng et al | Meta-analysis | 2016, international | 6 studies, (n=504) | Nasojejunal / nasoduodenal / jejunostomy vs parenteral nutrition in a central vein | Pulmonary complications refer to pneumonia and acute respiratory distress syndrome. No specific criteria given for each individual study. |
| Barlow et al | Multi-centre RCT | UK | n=121 | Early enteral nutrition or control | Chest infection – Abnormal CXR with pyrexia (>38°C) and WBC > 12,000 cells/ul and positive sputum. Respiratory failure - Presence of dyspnoea and respiratory rate >35/min or PaO_2_ <70 mm Hg. |
| Tian et al | Retrospective cohort | China | n=1400 | Jejunostomy vs Nasojejunal tube feeding | Pulmonary infection – no criteria given |
| Han-Geurts et al | RCT | The Netherlands | n=150 | Jejunostomy vs nasoduodenal tube feeding started on first day after surgery | Pneumonia – no criteria given |
| Shen et al | Meta-analysis | International | 13 studies, n=3736 | Patients with and without a feeding jejunostomy tube | Pulmonary complications – no definitions given |

CXR; Chest x-ray, PaO_2_; Partial pressure of oxygen, RCT; Randomised controlled trial, WCC; White cell count.

Table S9: There is no evidence that specific nutritional formulae reduce the incidence of pulmonary complications. (Grade D evidence; no recommendation)

| **Author** | **Study design** | **Origin and year** | **Sample size** | **Intervention** | **Outcome measures of pulmonary complications** |
| --- | --- | --- | --- | --- | --- |
| Matsuda et al | RCT | 2017, Japan | n=87 | Use of an enteral immune modulating diet (IMD) vs a standard enteral diet | The primary outcome, namely the change in the oxygenation status (PaO_2_/FIO_2_ ratio), was assessed at baseline and on PODs 1 to 4, 6, and 8.  Also measured incidence of pneumonia (undefined) |
| Moro et al | Retrospective analysis | 2016, Japan | n=74 | Low fat elemental formula vs standard fat containing polymeric formula administered enterally via jejunostomy | Incidence of chyle leak (milky fluid exceeding 500ml per day), duration of respirator use, and pneumonia (no specific criteria). |
| Aiko et al | Retrospective analysis | 2008, Japan | n=29 | Use of immuno enhanced diets containing omega-3 fatty acids, arginine and RNA vs a standard formula given enterally through jejunostomy | Rates of pneumonia and anastomotic leak given (no specific definitions for either). |

FiO_2_; Fraction of inspired oxygen, PaO_2_; Partial pressure of oxygen, POD; Postoperative day, RCT; Randomised controlled trial.

Table S10: We recommend chest physiotherapy and early mobilisation to reduce pulmonary complications. (Grade C evidence, strong recommendation).

| **Author** | **Study design** | **Origin and year** | **Sample size** | **Intervention** | **Outcome measures of pulmonary complications** |
| --- | --- | --- | --- | --- | --- |
| Lunardi et al | Retrospective Review | Brazil, 2011 | 70 | Chest physical therapy and including lung re-expansion and airway clearance and early mobilisation vs early mobilisation only. | Time under mechanical ventilation, use of Cephalosporin antibiotic use, need to return to mechanical ventilation.  Respiratory complications: Atelectasis – confirmed radiographically, pneumonia – radiographically, high WCC and high temperature, pleural effusion – radiographically. |
| Nakamura et al | Retrospective Review | Japan, 2008 | 184 | The respiratory physiotherapy included breathing exercises with pursed lips, huffing, coughing, abdominal respiration, and expectoration. Information was provided regarding the importance of early mobilization. | Retrospectively - Pulmonary complications were defined by the presence of bronchopneumonia and aspiration pneumonia as diagnosed by their radiologic, clinical, and microbiological features, as well as the presence of respiratory failure requiring ventilatory support. |
| Pasquina et al | Systematic Review | 2006, international | 13 trials (n=1441) | Various regimens vs none | Pneumonia (6 trials), atelectasis (9 trials), non-specific pulmonary complications (8 trials). Definitions varied between trials. Also Pa0_2_/Fi0_2_ ratios (5 trials). |
| Odor et al | Meta-analysis | 2020, international | 12 trials (n=1345) | Variety of physiotherapy regimens were tested  in the included studies. All had supervision of patients by a physiotherapist for at least three preoperative or postoperative days. (3 studies pre-operative, 8 studies post-op) vs no physiotherapy as standard. | Primary outcome - Postoperative pulmonary complications (PPCs) – defined as any respiratory infection, respiratory failure, pleural effusion, atelectasis, or pneumothorax.  Secondary outcomes - respiratory infections, atelectasis  All required definition by authors (varied). |
| Hanada et al | Retrospective | 2018, Japan | n=118 | Non comparative study as all patients underwent some form of physiotherapy. Comparing those that mobilised from POD 1 compared to those that had a delay. Reasons for delay included dyspnoea, arrhythmias, hypotension, nausea and vomiting and pt choice | PPCs were defined by review of medical notes  Atelectasis: seen on CXR or CT scan postoperatively  Pneumonia: fever, raised WCC and sputum microbiology as well as CXR findings requiring antibiotics. |
| Nascimento et al | Cochrane Review | 2014, international | 8 studies n=1160 | 4 trials (n=152) compared incentive spirometry (IS) with no treatment, 2 trials (n=194) compared IS with deep breathing, 2 trials (n=946) compared IS with other chest physiotherapy, | Respiratory failure – undefined  Pulmonary complications: atelectasis – radiographic diagnosis, pneumonia – radiography, respiratory symptoms with WCC and temperature. |

CT; Computerised tomography, CXR; Chest x-ray; FiO_2_; Fraction of inspired oxygen, PaO_2_; Partial pressure of oxygen, POD; Postoperative day, PPC; Post-operative pulmonary complication, WCC; White cell count.

Table S11: We do not recommend more than one thoracic drain to reduce pulmonary complications. (Grade C evidence; weak recommendation).

| **Author** | **Study design** | **Origin and year** | **Sample size** | **Intervention** | **Outcome measures of pulmonary complications** |
| --- | --- | --- | --- | --- | --- |
| Cai et al | Retrospective Cohort | 2018, China | n=50 | 2 thoracic drains (1 x CTD and 1 x transhiatal MD) vs only transhiatal MD | Pulmonary infection, pneumothorax, chylothorax – for all no specific diagnostic criteria given. Pain scoring – day 0, 1, 2 and 3 postoperatively. |
| De Pasqual et al | Retrospective Cohort | 2020, Italy | n=239 | 2 thoracic drains (1 x apical drain and 1 x AD) vs apical only | ‘Pulmonary complications’ – no specifics given aside from according to the ‘Esophagectomy Complications Consensus Group’ |
| Zheng et al | Retrospective Cohort | 2018, China | n=78 | Group A – ICD and mediastinal drain, Group B mediastinal drain, Group C Transhiatal drain (unspecified if vacuum) | VAS scores for pain and respiratory complications (pneumonia, atelectasis, pneumothorax, pleural effusions) no specifics were given for diagnostic criteria. |
| Asti et al | Retrospective Cohort | 2018, Italy | n=100 | 15-Fr Transhiatal Blake Drain attached to portable vacuum bulb compared to 19-Fr ICD attached to underwater seal and suction | Ketorolac requirement (mean), Respiratory complications given - atelectasis, pneumonia, left pleural effusion, pneumothorax, air leak in the underwater bottle, chyle leakage haemothorax – no specific criteria mentioned |
| Wang et al | Retrospective Cohort | 2020, China | n=108 | ICD vs transhiatal drain. Both 14-Fr placed with tip at cervical anastomosis | Pain scores based on VAS (7am, 11am, 3mp and 7pm), pleural effusion – drainage of volume > 800ml, pneumothorax was distance >3cm from apex of lung and ribcage, chest drain reinsertion due to effusion or pneumothorax and extubation time. Major pulmonary complications, pneumonia, ARDS and chylothorax had no specific diagnosis. |
| Bhandari et al | RCT | 2015, China | n=60 | Comparison of removal of drain if output < 50ml in 24 hours compared to < 250ml | Extubation time and pleural effusion. Both groups screened for effusion on day 3. No definition given for pleural effusion. |
| Yao et al | Retrospective Cohort | 2016, China | n=70 | Comparison of removal of drain if output < 150ml in 24 hours compared to < 300ml. | Pneumonia, pleural effusion and atelectasis confirmed by pre-removal of drain CXR. Also residual pneumothorax (any visible air between lung and chest wall on the CXR), subcutaneous emphysema and re-admission due to pleural effusion within 30 days. |
| Johansson et al | RCT | 1998, Sweden | n=101 | Active suction (15mmHg) vs passive drainage, via 28Fr CTD. | The degree (mm) and duration of pneumothorax determined until chest drain removal and subsequently. Pleural effusion graded from 0 to 3; grade 0, no effusion, grade 1;1-2cm effusion, grade 2; 2-8cm, grade 3; more than 8cm and were documented for both sides. Atelectasis (no definition given), |
| Bull et al | Systematic Review | 2021, UK | 27 studies, n=2564 | Studies reporting outcomes for different types or uses of thoracic drainage, or outcomes related to drains after trans-thoracic oesophagectomy were included. Studies were collated into domains based on variations in number, position, type, removal criteria, diagnostic use and complications of drains. | N/A |

AD; Anastomotic drain, ARDS; Acute respiratory distress syndrome, CTD; Closed thoracic drainage, ICD; Intercostal drain, MD; Mediastinal drain, RCT; Randomised controlled trial, VAS; Visual analogue score.

Table S12: We recommend enhanced recovery pathways to reduce pulmonary complications. (Grade C evidence; strong recommendation).

| **Author** | **Study design** | **Origin and year** | **Sample size** | **Intervention** | **Outcome measures of pulmonary complications** |
| --- | --- | --- | --- | --- | --- |
| Zhang et al | RCT | 2017, China | n=94 | Non-enhanced vs ERAS (enhanced recovery after surgery). Traditional included NGT decompression and fasting for 7 days. FTS included oral food POD 1, early mobilisation and TPN for 3 days. | Pneumonia – no definition |
| Li et al | RCT | 2017, China | n=110 | Non-enhanced vs ERAS. ERAS included patient education RE ERAS, preoperative nutrition evaluation, early ambulation, enteral nutrition via jejunostomy 6-12 hour post-op, oral nutrition from POD 7 WSS | Frequency of sputum suction with a bronchoscope and pulmonary infection. The criteria for sputum suction included lack of effective cough, thick and heavy sputum, atelectasis showed by X-rays or CT images The presence of pneumonia is defined by new lung infiltrate plus clinical evidence that the infiltrate is of an infectious origin. Length of hospital stay. |
| Chen et al | RCT | 2016, China | n=276 | ERAS included patient education RE ERAS, no routine use of NGT, early mobilisation sit out from POD 1, epidural analgesia, jejunostomy feeding POD 1, oral drink from POD 4 if WSS clear. | Pneumonia and ARDS (no definition given), numerical pain scale. Inflammatory markers within blood. |
| Zhao et al | RCT |  | n=68 | ERAS included patient education RE ERAS, no routine use of NGT, early mobilisation sit out from POD 1, epidural analgesia, jejunostomy feeding POD 1, oral drink from POD 4 if WSS clear. | Pneumonia (no definition given), incision pain score, postoperative hospital stay |
| Triantafyllou et al | Meta-analysis | 2020, Greece, India, USA. | n=1133 (8 studies) | Multiple ERAS regimens compared vs standard care in oesophageal surgery | Pulmonary Complications – no specific criteria given. |
| Huang et al | Meta-analysis | 2020, China | 15 studies, n=1496 | Multiple ERAS regimens compared vs standard care in upper gastrointestinal surgery (oesophagus, stomach and duodenum) | Lung infection – no specific definition given for individual studies. |
| Markar et al | Meta-analysis | 2015, International | 9 studies, n=1240 | ERAS via conventional pathway, no definitions given for included ERAS pathways. | Pulmonary complications (including pneumonia, pneumothorax and respiratory failure). No definitions given. |

ARDS; Acute respiratory distress syndrome, ERAS; Enhanced recovery after surgery, NGT; Naso-gastric tube, POD; Postoperative day, RCT; Randomised controlled trial, TPN; total parenteral nutrition, WSS; Water soluble swallow.

**Areas of further research with research questions**

Table S13: What is the recommended surgical position for the patient to be placed in during oesophagectomy procedures?

| **Study** | **Study design** | **Origin and year** | **Sample size** | **Intervention** | **Outcome measures of pulmonary complications** |
| --- | --- | --- | --- | --- | --- |
| Miura et al | Retrospective cohort study | 2019,  Japan | 200 | 1. Thoracoscopic oesophagectomy in prone position (TEP) 120 pts  2. Thoracoscopic oesophagectomy in lateral position (TEL) 80pts | Primary outcome: OS and PFS at 5 years. Secondary outcomes included pulmonary complications  More pulmonary complications in TEL - defined as pneumonia and atelectasis with antibiotics  Before matching: TEL (30.0%) vs TEP (16.7%)  After matching: TEL (30.8%) vs TEP (15.4%) p<0.05 |
| Kuwabara et al | Retrospective cohort study | 2018,  Japan | 142 | 1. Thoracoscopic oesophagectomy in left decubitus position (LP) 72 pts  2. Thoracoscopic oesophagectomy in prone position (TEP) 70pts | Outcomes: 1. Operation time and blood loss, 2. Operative morbidity, 3. Mortality, 4. LOS, 5. No. of LNs dissected, 6. OS, 7. Recurrence  Pulmonary complications  - defined as fever greater than 38 °C, with graphic evidence of pneumonia or atelectasis  Lower in the TEP group (7%) than in the LP group (30%) p<0.01 |

LOS; Length of stay, LN; lymph node, LP; Left decubitus position, OS; Overall survival, PFS; Progression free survival; TEL; Lateral position, TEP; Prone position.

Table S14: What is the evidence for the use of beta agonists in oesophagectomy procedures?

| **Study** | **Study design** | **Origin and year** | **Sample size** | **Intervention** | **Outcome measures of pulmonary complications** |
| --- | --- | --- | --- | --- | --- |
| **Perkins et al**  **(BALTI trial)** | Multicentre RCT | 2013  UK | 179 (salmeterol)  183 (placebo) | Perioperative B agonists | 1. ALI within 72hrs of surgery: acute onset of bilateral infiltrates on CXR and hypoxemia  (PaO_2_/FIO_2_ ratio of 300 mm Hg) in the absence of clinical evidence of left atrial hypertension  2. No definition for pneumonia  3. Inflammatory protein levels |

ALI; Acute lung injury, CXR; Chest x-ray, FiO_2_; Fraction of inspired oxygen, PaO_2_; Partial pressure of oxygen, RCT; Randomised controlled trial.

Table S15: What is the evidence for the use of neutrophil elastase inhibitors in oesophagectomy procedures?

| **Study** | **Study design** | **Origin and year** | **Sample size** | **Intervention** | **Outcome measures of pulmonary complications** |
| --- | --- | --- | --- | --- | --- |
| Wang et al | SR of 13 studies (5 RCTs, 8 nRCTs) | 2015 | - | Sivelestat (a  neutrophil elastase inhibitor) | Pneumonia  Duration of ventilation  Acute lung injury |

RCT; Randomised controlled trial.

Table S16: What is the recommended mode of ventilation (one lung vs two lung) during oesophagectomy procedures?

| **Study** | **Study design** | **Origin and year** | **Sample size** | **Intervention** | **Outcome measures of pulmonary complications** |
| --- | --- | --- | --- | --- | --- |
| Saikawa et al | Prospective | 2013  Japan | 14 | Two lung ventilation during artificial pneumothorax (AP) in prone position  Comparator: prone position prior to AP | 1. Mean P/F ratio during AP 244.4 mmHg, p < 0.05  2. PaCO_2_ rose a mean of 7 mmHg during to 48.3 mmHg during AP, p < 0.05  3. Intra-op peak airway press. (Apmax) increased by mean 4.2 cmH_2_O (p <0.05) to peak 18-20 cmH_2_O throughout AP.  4. No major changes in tidal volume. Non-significant. |
| Nomura et al | Single-centre, retrospective study | 2020  Japan | 119 | One lung ventilation  Vs  Two lung ventilation | 1. Respiratory complications & function not defined  2. TLV group had a significant increase in PaO_2_/ FiO_2_ ratio on POD5 and POD7  3. CRP on POD7 lower in TLV 7 mg/dL vs 10 mg/dL (p <0.05)  4. Reintubation: 18.3% in OLV vs 8.3% in TLV, p = 0.18  5. Resp complications: 23.9% in OLV vs 16.7% in TLV, p = 0.37 |

AP; Artifical pneumothorax, FiO_2_; Fraction of inspired oxygen, OLV; One-lung ventilation, PaO_2_; Partial pressure of oxygen, PaCO_2_; Partial pressure of carbon dioxide, POD; Postoperative day, TLV; Two-lung ventilation.
